# Supplementary material for: Bidimensional lamellar assembly by coordination of peptidic homopolymers to platinum nanoparticles
Source: Nat Commun. 2020 Apr 28;11:2051. doi: 10.1038/s41467-020-15810-y (PMC7188844; doi:10.1038/s41467-020-15810-y)
Supplement: Supplementary file 1 — Supplementary Information [file 41467_2020_15810_MOESM1_ESM.pdf]

# Supplementary information

# Bidimensional lamellar assembly by coordination of peptidic polymers to platinum nanoparticles

Ghada Manai,[a,b,ǂ] Hend Houimel,[a,ǂ] Mathilde Rigoulet,[a] Angélique Gillet,[a] Pier-Francesco Fazzini,[a] Alfonso Ibarra,[c] Stéphanie Balor,[d] Pierre Roblin,[e] Jérôme Esvan,[f] Yannick Coppel,[b] Bruno Chaudret,[a] Colin Bonduelle,[b,g,*] and Simon Tricard[a,*]

[a] *Laboratoire de Physique et Chimie des Nano-Objets, INSA, CNRS, Université de Toulouse, Toulouse, France*

[b] *Laboratoire de Chimie de Coordination, CNRS, Université de Toulouse, Toulouse, France*

[c] *Instituto de Nanociencia de Aragón, Universidad de Zaragoza, Zaragoza, Spain*

[d] *Plateforme de Microscopie Électronique Intégrative, Centre de Biologie Intégrative, CNRS, Université de Toulouse, Toulouse, France*

[e] *Laboratoire de Génie Chimique, Fédération Fermat, INPT, CNRS, Université de Toulouse, Toulouse, France*

[f] *Institut Carnot – Centre Inter-universitaire de Recherche et d'Ingénierie des Matériaux, INP-ENSIACET, CNRS, Université de Toulouse, Toulouse, France*

[g] *Laboratoire de Chimie des Polymères Organiques, Université de Bordeaux, CNRS, Bordeaux INP, Pessac, France*

[*] *Corresponding authors: colin.bonduelle@enscbp.fr, tricard@insa-toulouse.fr*

[,ǂ] *These authors contributed equally to this work*

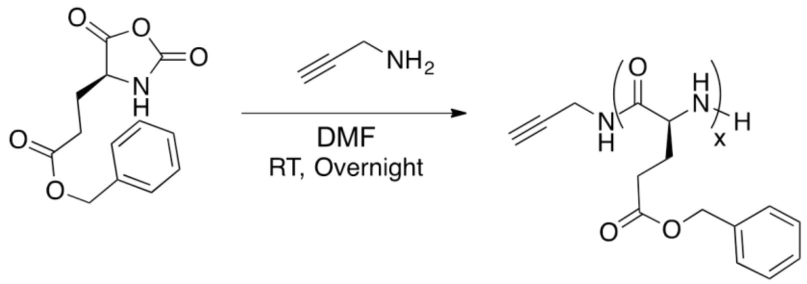


**Supplementary Figure 1. PBLG synthesis.** Synthetic scheme depicting poly(γ-benzyl-*L*-glutamate) (PBLG) polymers preparation. In the present study, x = 28 (**PBLG1**), 69 (**PBLG2**), 120 (**PBLG3**), 217 (**PBLG4**), 481 (**PBLG5**).

| Sample | NCA | Propargyl -amine | Theoretical Dp [a] | Dp from $^{1}$H NMR [b] | Mn PBLG from SEC (Dp) [c] | PDI PBLG from SEC [c] |
|---|---|---|---|---|---|---|
| **PBLG1** | 2g | 16 µL | 30 | 25 | 6200 (28) | 1.23 |
| **PBLG2** | 2g | 8 µL | 60 | 59 | 15100 (69) | 1.21 |
| **PBLG3** | 2g | 4 µL | 120 | 92 | 26500 (120) | 1.25 |
| **PBLG4** | 2g | 2 µL | 240 | 171 | 47500 (217) | 1.25 |
| **PBLG5** | 2g | 1.2 µL | 400 | 373 | 105000 (481) | 1.33 |

**Supplementary Table 1**. Chemical characterizations of the PBLG polymers. [a] Theoretical degree of polymerization ([M]/[I]); [b] Number average molar mass (Mn) determined by $^{1}$H NMR (see methods). [c] Absolute number average molar mass (Mn) and dispersity (Đ) determined by SEC using a multi-angle static light scattering detection (SEC in DMF (+LiBr), see methods).

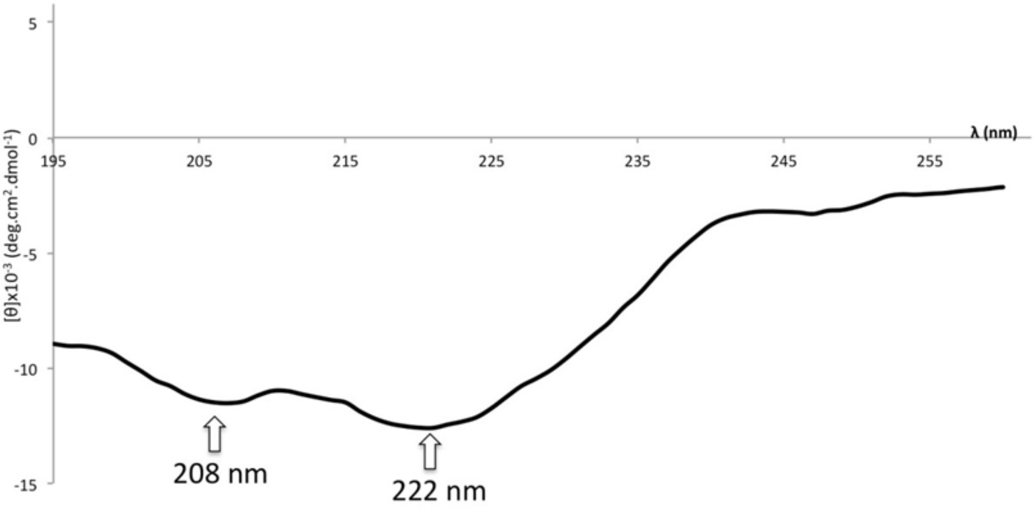


**Supplementary Figure 2. Circular dichroism spectrum of PBLG4.** The specific negative bands at 208 and 222 nm confirm the α-helix conformation of the polymer in THF. It is to note that the resolution of the CD measurement is strongly limited by the UV absorbance of the THF and implies the use of a specific set up before analysis (see methods).

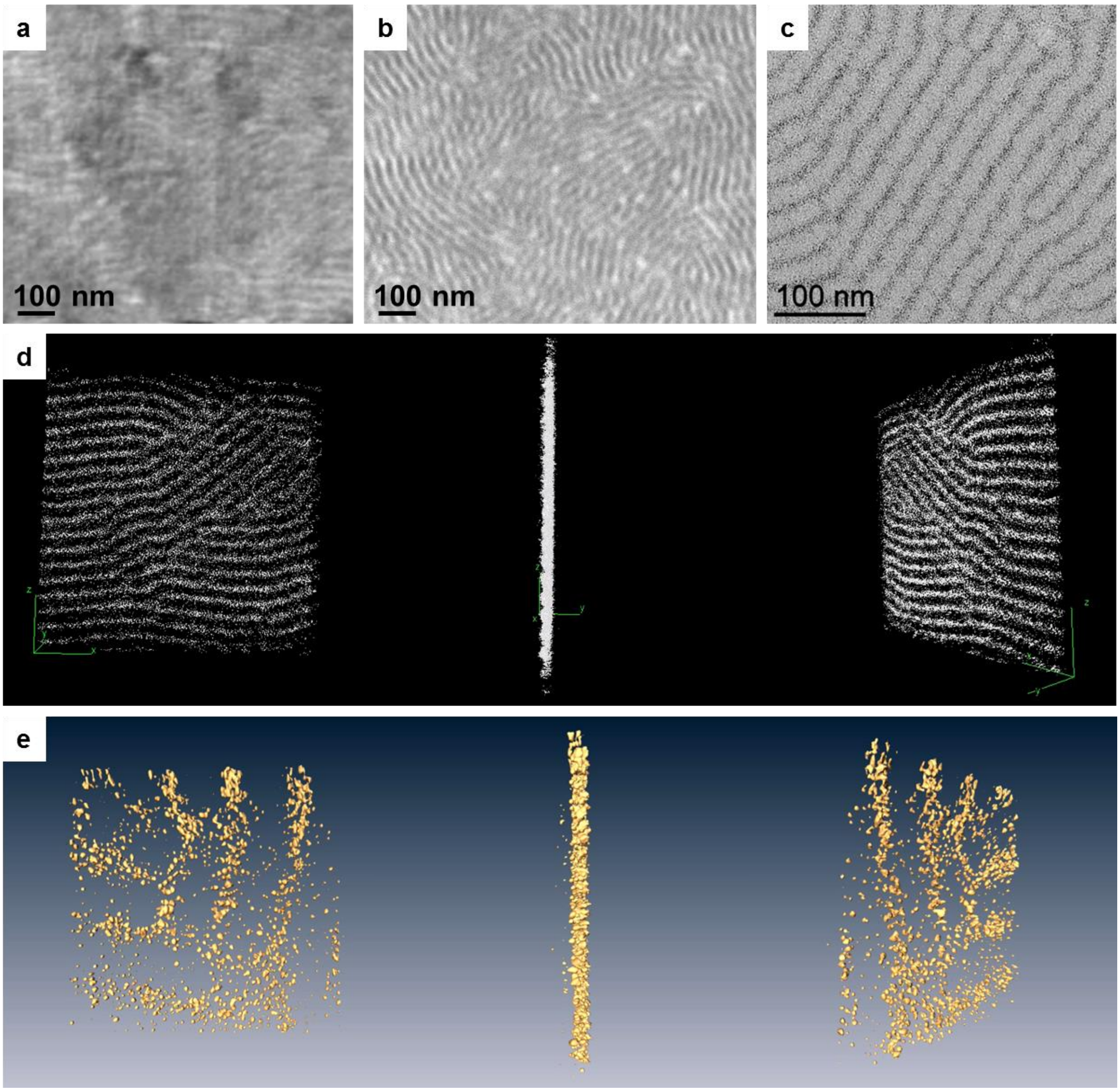


**Supplementary Figure 3. Imaging of the lamellar structure of self-assembly of platinum nanoparticles with PBLG4, by different techniques: a**, Atomic Force Microscopy on silicon; **b**, Scanning Electron Microscopy on silicon; **c**, Low-magnification Transmission Electron Microscopy on carbon. **d**,**e**, Tomographv 3D reconstructions at different viewing directions: **d**, at low magnification and **e**, at high magnification.

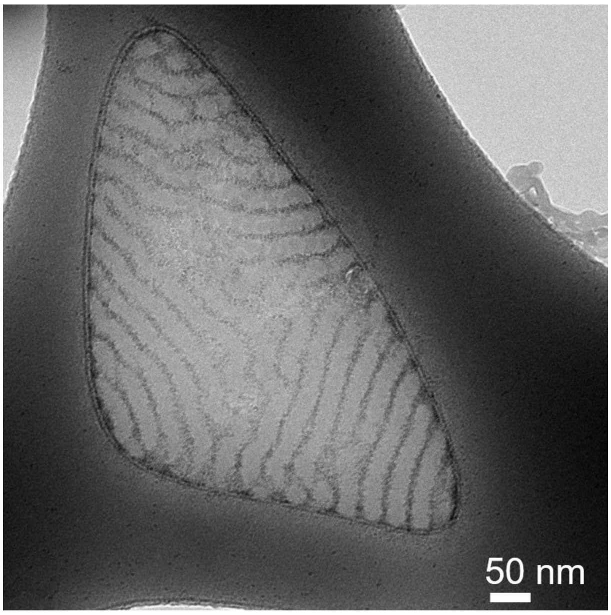


**Supplementary Figure 4. Cryo-TEM imaging.** Cryo-TEM imaging of self-assembly of platinum nanoparticles with **PBLG4**. Fast freezing of the THF solution confirms the presence of the lamellar structures in solution.

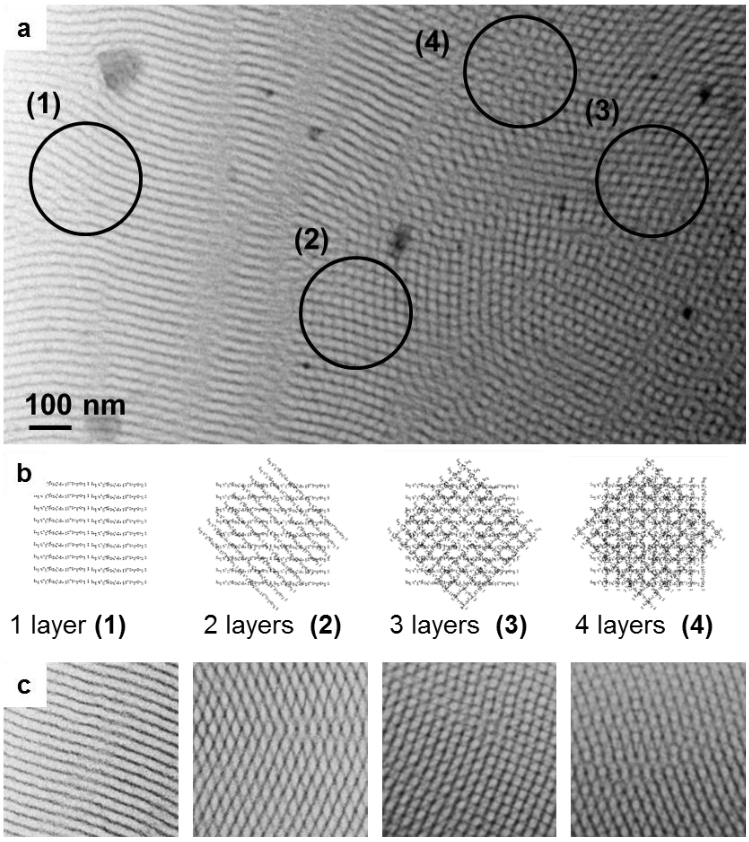


**Supplementary Figure 5. Moiré patterns of lamellar self-assemblies of platinum nanoparticles with PBLG4. a**, TEM image. Up to four 2D lamellar structures superimpose on each other to form specific Moiré patterns: zone (1) single layer – no Moiré, zone (2) two layers, zone (3) three layers, zone (4) four layers. **b**, Corresponding schematics for each zone, where the scheme of n layers is obtained by superimposing the scheme of 1 layer to the scheme of n-1 layers with a tilt of 45°. **c**, Picture of the same layer superimposed to itself 0, 1, 2, and 3 times after rotation; the same Moiré patterns as in zones (1) to (4) of Supplementary Fig. 5a were then artificially reconstructed.

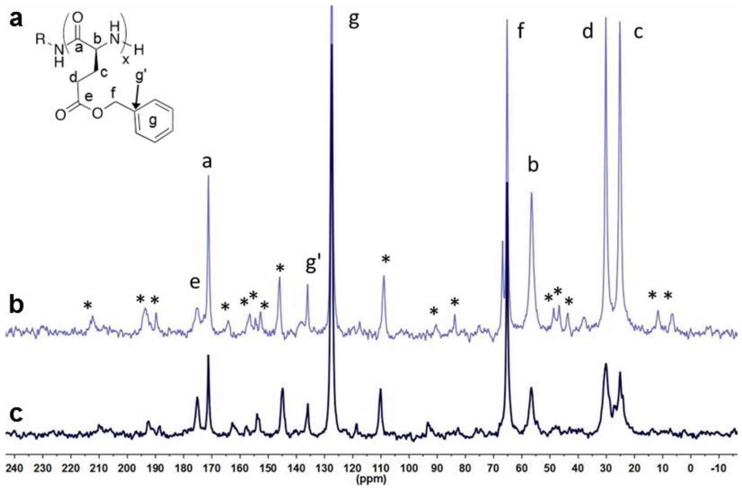


**Supplementary Figure 6. $^{13}$C HR-MAS NMR study of PBLG4. a**, Chemical structure of PBLG, and carbon position labelling (here x = 217). **b**, Spectrum of self-assembly of platinum nanoparticles with **PBLG4**. **c**, Spectrum of **PBLG4** alone. The spectra were obtained in THF at 1,8 kHz (* = rotation bands). The appearance of new rotation bands and the narrowing of the polymer peaks show a rigidifying of the polymer structures in the self-assembly.

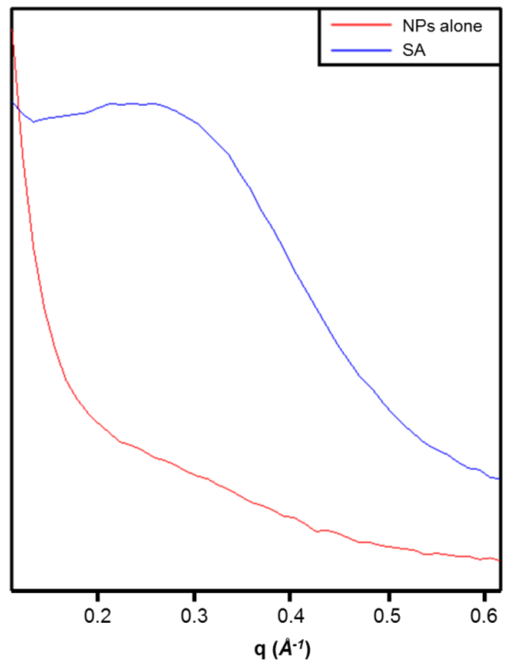


**Supplementary Figure 7. Interparticle distance.** Small angle X-ray scattering patterns for platinum nanoparticles alone (without PBLG – NPs alone) and for the self-assembly of platinum nanoparticles with **PBLG4** (SA). The nanoparticles alone don't show any specific signal in addition to the continuous background, whereas the self-assembly shows a broad peak, which corresponds to a specific correlation distance between the nanoparticles equal to 2.5 nm ($2\pi / q_{max}$).

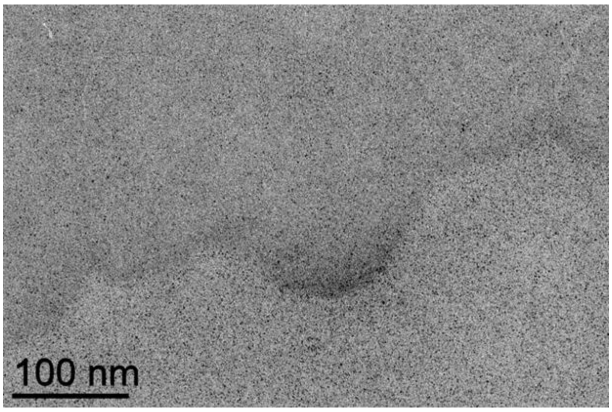


**Supplementary Figure 8. Absence of self-assembly in high quantity of PBLG.** TEM micrograph of mixing of platinum nanoparticles with 5eq. of **PBLG4**.

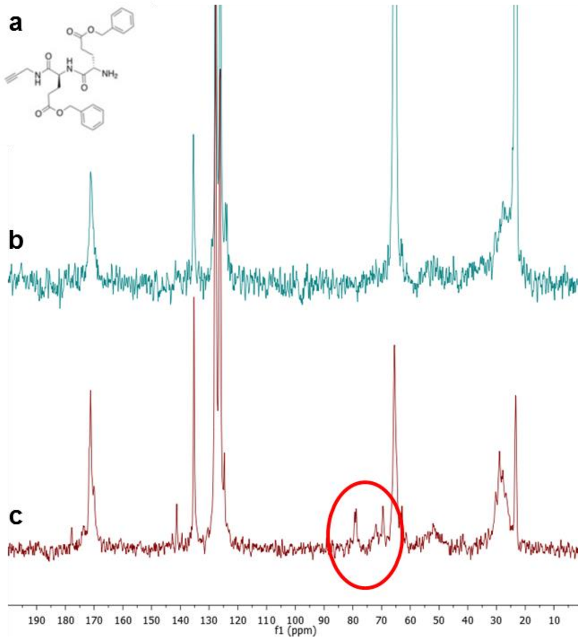


**Supplementary Figure 9. $^{13}$C solid-state MAS NMR study of DBLG. a**, Chemical structure of **DBLG**. **b**, Spectrum of self-assembly of platinum nanoparticles with **DBLG**. **c**, Spectrum of **DBLG** alone. The spectra were obtained at 10 kHz. The disappearing of the peaks at 72 and 80 ppm is in agreement with the coordination of the alkyne moiety of the polymer to the nanoparticle surface in the self-assembly.

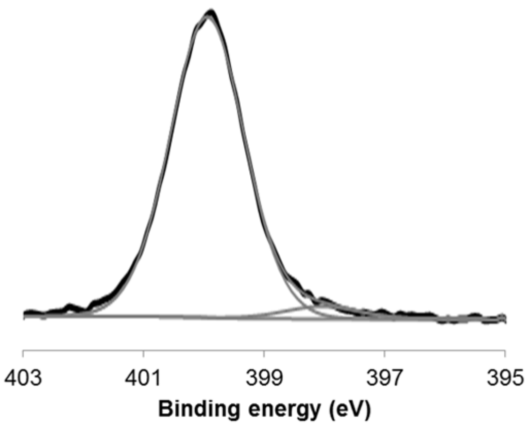


**Supplementary Figure 10. Reduced component at 5 eq.** XPS spectrum at the N*1s* edge of mixing of platinum nanoparticles with 5eq. of **PBLG4** (4% of contribution at 398 eV). We start to observe the appearance of the reduced contribution of nitrogen at 5 eq.

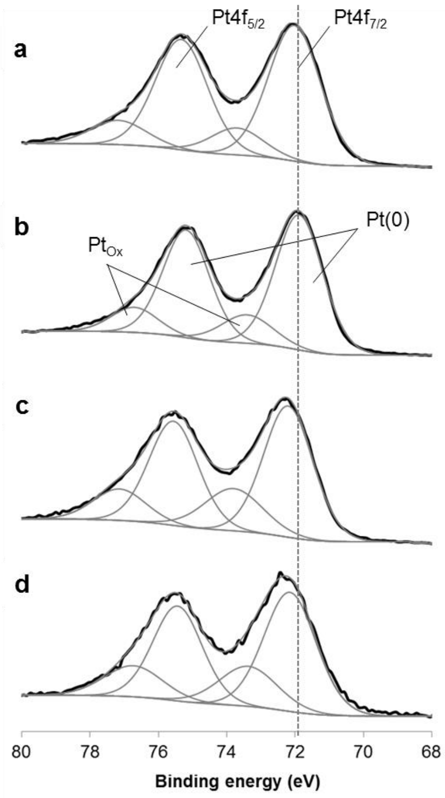


**Supplementary Figure 11. Relative oxidation of platinum upon addition of PBLG.** XPS spectra at the Pt*4f* edge of assemblies between platinum nanoparticles and **PBLG4** at: a, 0.05 eq. (18% of oxidized contribution); b, 0.5 eq. (19% of oxidized contribution); c, 1 eq. (26% of oxidized contribution); d, 5 eq. (28% of oxidized contribution). The progressive increase of the oxidized contribution at ~73.4 and ~76.7 eV from 0.05 eq. to 5 eq. and the slight increase of the global Pt*4f* binding energies (a dashed line is added as a guide for the eye) confirmed an increase of the relative platinum oxidation at the NP surface upon addition of PBLG.

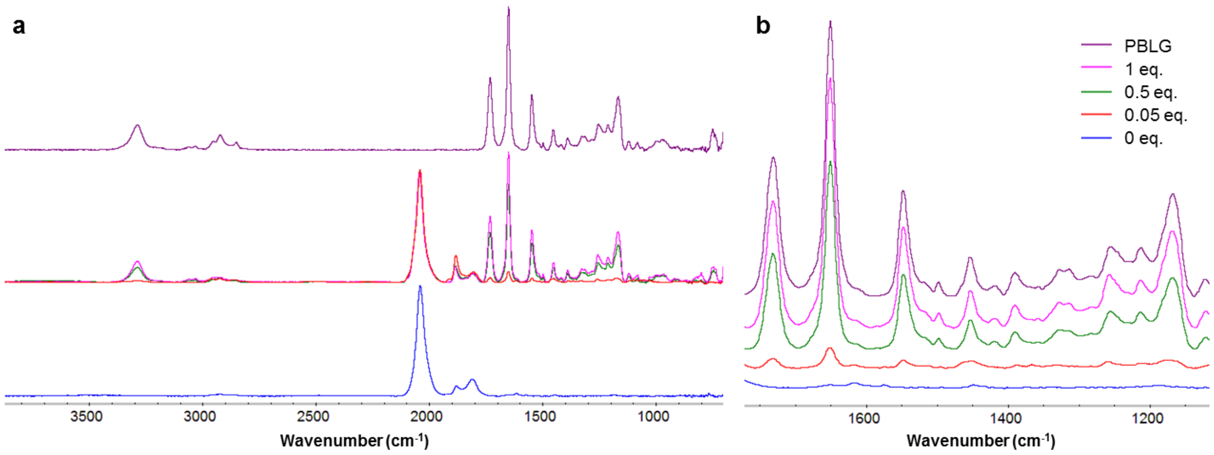


**Supplementary Figure 12. FT-IR spectra. a**, Full range FT-IR spectra of **PBLG4** alone and of the self-assemblies between platinum nanoparticles and **PBLG4** (at 0 eq., 0.05 eq., 0.5 eq., and 1 eq.) and **b**, zoom on the **PBLG4** fingerprint region – the spectra are shifted for clarity. The esters (1770 $cm^{-1}$) and amide (1770 $cm^{-1}$ and 1770 $cm^{-1}$) vibrations are not affected by the NP addition. The spectra are normalized on the signal of the CO vibration around 2040 $cm^{-1}$.

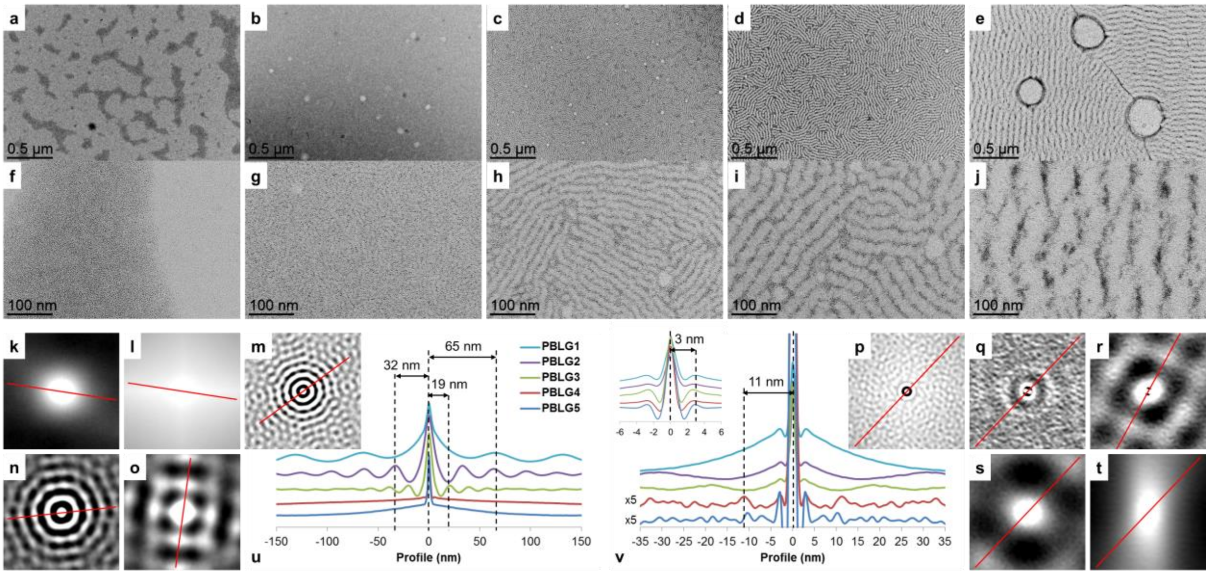


**Supplementary Figure 13. Effect of the degree of polymerization – image analysis.** TEM micrographs at two scales of self-assemblies between platinum nanoparticles and: **a**, **f**, **PBLG1**; **b**, **g**, **PBLG2**; **c**, **h**, **PBLG3**; **d**, **i**, **PBLG4**; and **e**, **j**, **PBLG5**. Autocorrelation images of : **k**, a; **l**, b; **m** c; **n**, d; **o**, e; **p**, f; **q**, g; **r**, h; **s**, i; **t**, j. **u**, Profiles represented on images k, l, m, n, and o. **v**, Profiles represented on images p, q, r, s, and t. (Inset : zoom on the central region of the profile). Systematic image analysis gives the average periodicity of the lamellas as shown on Fig. 4f and Supplementary Table 2.

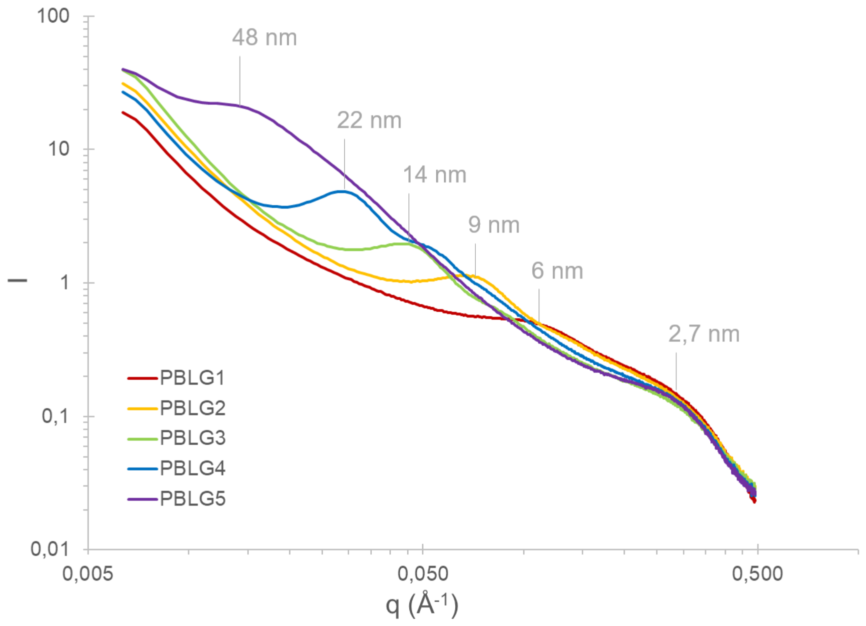


**Supplementary Figure 14. SAXS measurements.** SAXS measurements on self-assemblies between platinum nanoparticles and **PBLG1**, **PBLG2**, **PBLG3**, **PBLG4**, and **PBLG5**. The SAXS curves show the presence of multiple correlation peaks. For all samples, the large peak around 0.23 $Å^{-1}$ gives an average distance of 2.7 nm corresponding to the correlation distance between the nanoparticles, as already observed (Supplementary Fig. 7). The presence of one peak (for **PBLG1** and **PBLG2)** or two peaks (for **PBLG2**, **PBLG3** and **PBLG4**) in the first part of the curve comes from interactions at medium distances, and proves the presence of the lamellae in the macroscopic sample. The corresponding periodicities deduced from the position of the first peak are represented on the curves and reported in Supplementary Table 2. The second peak, when distinguishable, corresponds to the harmonic at a double *q* value, confirming the spacing is constant at higher distances than a single period. The SAXS measurements confirmed the increasing of the average inter-lamellar distance with the degree of polymerization. The broadness of the peaks reflects a distribution of distances, as observed by TEM imaging.

| Periodicity (nm) | TEM | SAXS |
|---|---|---|
| **PBLG1** | - | 6 |
| **PBLG2** | 11 | 9 |
| **PBLG3** | 19 | 14 |
| **PBLG4** | 32 | 22 |
| **PBLG5** | 65 | 48 |

**Supplementary Table 2. Comparison of the periodicities of the self-assemblies obtained by TEM (Supplementary Fig. 13) and SAXS (Supplementary Fig. 14) measurements. S**tructural and microscopy analyses gave coherent trends, where the periodicity of the lamellae increased with the degree of polymerization of the polymer. Differences of absolute values can be explained by the beginning of precipitation occurring during the SAXS measurements (and thus to a compression of the system due to the solvent exclusion). On the contrary, sample preparation for electron microscopy corresponds to a quenching of the system in solution (as the solvent is rapidly absorbed by a filter paper during the TEM grid preparation). The periodicity for the cryo-TEM images with **PBLG4** was found to be 31 nm (Supplementary Fig. 4), in line with the TEM measurements.

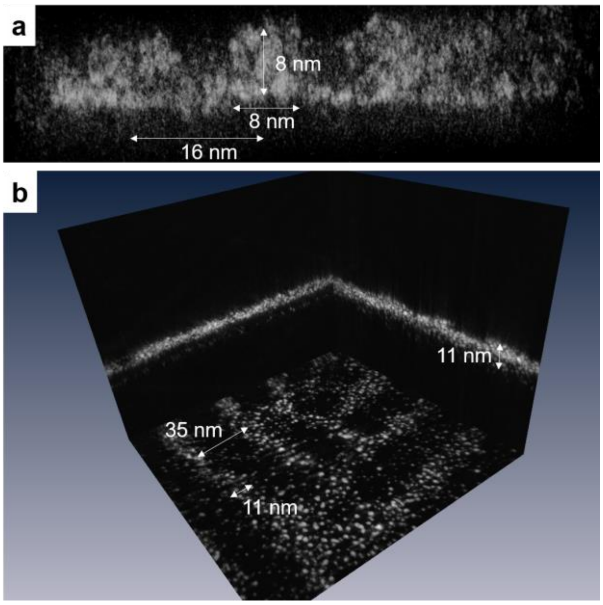


**Supplementary Figure 15. Geometrical analysis of tomography reconstructions.** Analysis of tomography reconstruction of self-assemblies between platinum nanoparticles and: **a**, **PBLG3** and **b**, **PBLG4** confirmed similar thicknesses and width of the NP containing zones. The local periodicity is coherent with average values obtained by systematic image analysis (Fig. 4, Supplementary Fig. 13, and Supplementary Table 2).

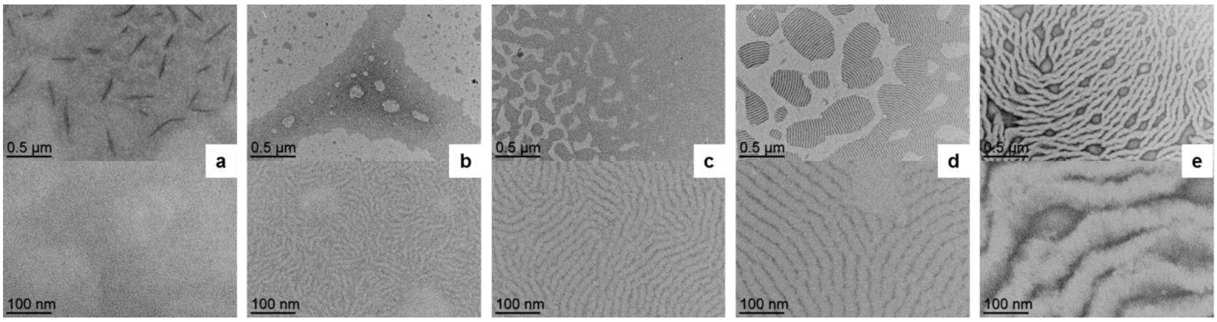


**Supplementary Figure 16. Effect of the degree of polymerization.** TEM micrographs at two scales of self-assemblies, keeping the polymer on nanoparticle ratio constant between platinum nanoparticles and **a**, **PBLG1** (0.06 eq.). **b**, **PBLG2** (0.16 eq.). **c**, **PBLG3** (0.28 eq.). **d**, **PBLG4** (0.50 eq.). **e**, **PBLG5** (0.81 eq.). Similar structures are obtained by keeping the monomer units per nanoparticle (Supplementary Fig. 13a to j) or the polymer number per nanoparticle constant.

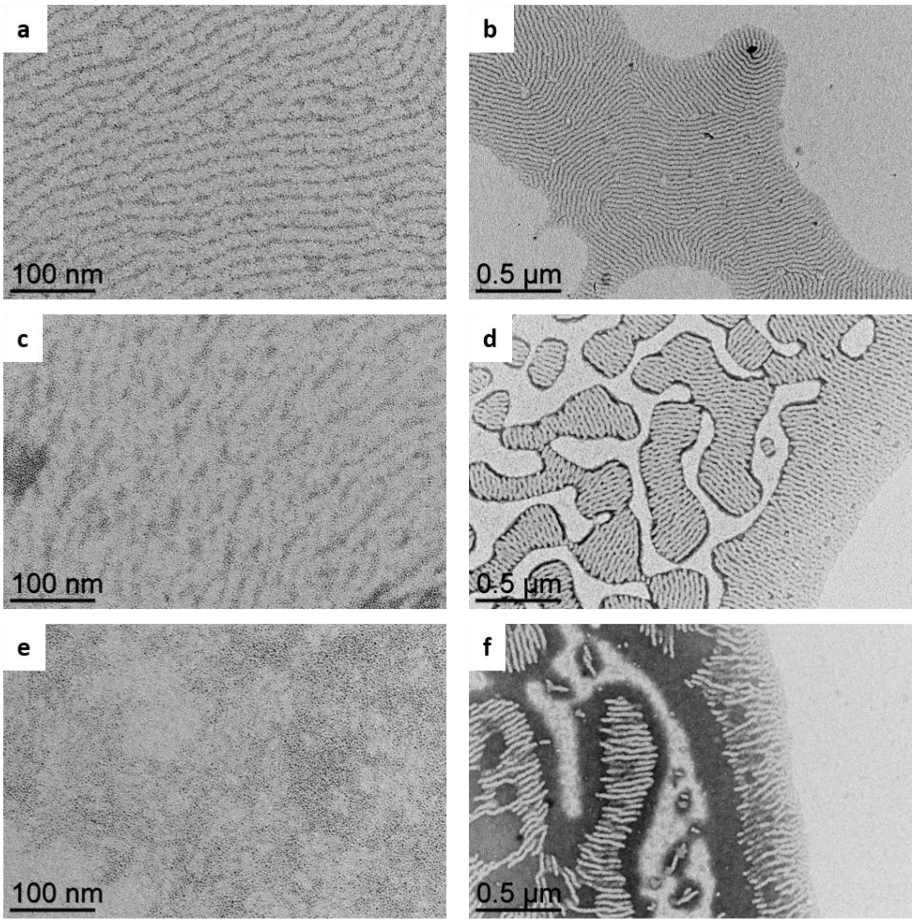


**Supplementary Figure 17. First steps of self-assembly.** TEM micrographs of self-assemblies between platinum nanoparticles and **a**, **c**, **e**: **PBLG3**; **b**, **d**, **f**: **PBLG4**: **a**, **b**: after 2 h of reaction. **c, d**: after 5 s of reaction. **e, f**: after a fraction of second of reaction – the nanoparticles are drop-casted on pre-deposited PBLG. The assembly behavior was comparable at the two different Dp (see main text).

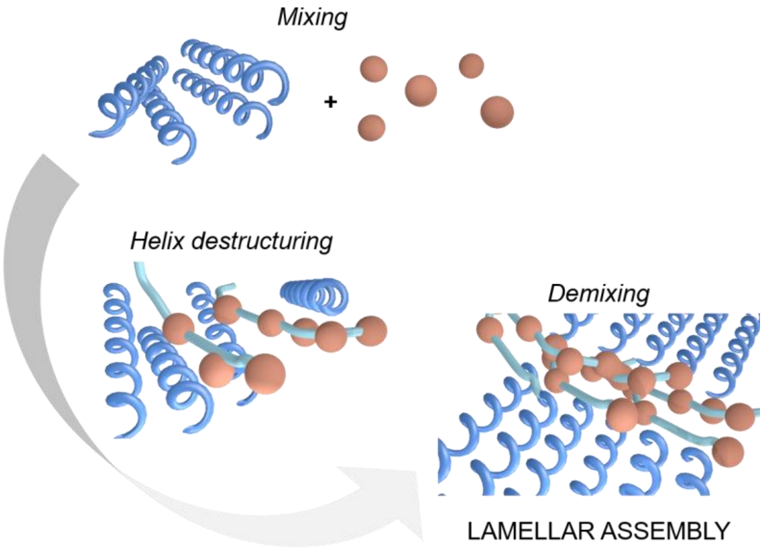


**Supplementary Figure 18. Scheme of the self-assembly process.** α-helices of PBLG tend to spontaneously align in THF solution. Upon mixing with platinum nanoparticles, some part of them are destructured thanks to interaction with their amide functional groups and the nanoparticle surface, and form random coils that interact with an average of 6-7 nanoparticles. Demixing between such a hybrid phase and the excess of PBLG in α-helices led to the alternation of lamellae, stabilized by the coordination of the alkyne terminal group of the polymer to the surface of accessible nanoparticles.
